# Identification of *MACC1 *as a novel prognostic marker in hepatocellular carcinoma

**DOI:** 10.1186/1479-5876-9-166

**Published:** 2011-09-29

**Authors:** Jiliang Qiu, Pinzhu Huang, Qian Liu, Jian Hong, Binkui Li, Canliang Lu, Li Wang, Jianping Wang, Yunfei Yuan

**Affiliations:** 1 State Key Laboratory of Oncology in South China/Department of Hepatobiliary Oncology, Sun Yat-sen University Cancer Center, Guangzhou, China; 2 Department of Surgery, the First Affiliated Hospital, Gannan Medical College, Ganzhou, China; 3 Department of General Surgery, Beijing Shijitan Hospital, Beijing, China; 4 Department of Surgery, the Sixth Affiliated Hospital, Sun Yat-sen University, Guangzhou, China

**Keywords:** MACC1, c-MET, hepatocellular carcinoma, prognosis

## Abstract

**Background:**

Metastasis-associated in colon cancer-1 (*MACC1*) is a newly identified gene that plays a role in colon cancer metastasis through upregulation of c-MET proto-oncogene (c-MET). However, the value of *MACC1 *as a potential biomarker for hepatocellular carcinoma (HCC) remains unknown.

**Methods:**

*MACC1 *mRNA expression in 128 HCC tissues was examined by quantitative polymerase chain reaction. To show the potential correlation of *MACC1 *and c-MET, c-MET was also analysed.

**Results:**

*MACC1 *was more highly expressed in HCC than in non-HCC tissues (*P *= 0.009). High *MACC1 *expression was significantly increased in cases with high alpha fetoprotein (AFP) (*P *= 0.025). A positive correlation was found between *MACC1 *and c-MET mRNAs (r = 0.235, *P *= 0.009). Both univariate and multivariate analyses revealed that *MACC1 *expression was associated with overall survival (OS) and disease-free survival (DFS). Moreover, stratified analysis showed that tumour-node-metastasis (TNM) stage I patients with high *MACC1 *levels had shorter OS and DFS than those with low *MACC1*.

**Conclusions:**

*MACC1 *may identify low- and high-risk individuals with HCC and be a valuable indicator for stratifying the prognosis of TNM stage I patients. *MACC1 *may serve as a novel biomarker for HCC.

## Background

Hepatocellular carcinoma (HCC) is one of the most common solid tumours and prevalent fatal cancers worldwide, especially in East Asia and Sub-Saharan Africa [1,2]. Recently, HCC mortality rate has increased faster than the mortality rates for any other leading cancers in the United States [3,4]. Surgical resection or liver transplantation offers the chance of a cure, but only 30-40% of HCC patients are eligible for curative treatments, even in developed countries [5]. The HCC recurrence rate is as high as 54% at 5 years, even in early-stage HCC after radical resection [6,7]. Survival may vary widely among HCC patients with the same clinicopathologic features, which is most likely attributable to the heterogeneity of the biological behaviour of tumour cells [8,9]. Although recent studies have unravelled some aberrantly expressed genes contributing to different prognoses in HCC, the molecular markers that help to predict early recurrence and serve as potential targets remain limited.

The importance of understanding the molecular biology of HCC has recently gained considerable attention, as molecular targeting therapy has shown encouraging results for many malignancies [10,11]. The key signal transduction pathways implicated in the pathogenesis of liver cancer include the PI3K/Akt/mTOR pathway [12], Wnt/β-catenin signalling cascade [13], and HGF/c-MET pathway [14]. Recently, numerous disorders related to deregulation of the HGF/c-MET axis have been reported [15,16]. Aberrant activity of c-MET elicits multiple cellular responses regulating cell morphogenesis, migration, and breakdown of the extracellular matrix. Dysregulation of c-MET is common in HCC [17], although the exact mechanisms of this pathway in the carcinogenesis of HCC are still under investigation. As compounds that target the HGF/c-MET pathway are developed, new treatments for c-MET-triggered malignancies may be designed and the sensitivity of molecular-targeted drugs that are in clinical use may be improved [18,19].

Poor prognosis of HCC is often associated with a high potential of vascular invasion and metastasis [20,21]. c-MET is one of the key players in the processes of dissociation, angiogenesis, and migration of tumour cells in HCC [17]. Metastasis-associated in colon cancer-1 (*MACC1*), a new gene associated with colon cancer in primary and metastatic carcinomas, promotes tumour cell growth as well as the development of distant metastases [22]. Overexpression of MACC1 induces downstream activation of HGF/c-MET and facilitates metastasis of colon cancer, while silencing of MACC1 leads to reduced tumour proliferation, decreased cell migration, and a lack of new metastases, indicating the importance of MACC1 in the phases of cancer progression. Although MACC1 has been studied in colon carcinoma, little is known about its role in HCC. To address this issue, we evaluated the expression of *MACC1 *mRNA to determine whether *MACC1 *expression is of prognostic significance in HCC. Given the tight correlation between *MACC1 *and c-MET in colon cancer, we also examined the expression level of c-MET mRNA to determine whether such correlation exists in HCC.

## Methods

### Cell lines

Four human HCC cell lines, specifically HepG2, SMMC-7721, MHCC-97H, and MHCC-97L, and one immortalised nontumourigenic normal human hepatocyte cell line, L-02, were used to screen the expression of *MACC1*. The hepatoma cell line HepG2 was purchased from the American Type Culture Collection (Manassas, VA). L-02, SMMC-7721, MHCC-97H and MHCC-97L were obtained from the Type Culture Collection of the Chinese Academy of Sciences (Shanghai, China) and maintained under recommended culture conditions. Cells were grown at 37°C in a humidified incubator containing 5% CO_2_. RNA was extracted from exponentially growing cells.

### Study population and sample collection

A total of 128 pairs of HCC tissues and adjacent nontumorous liver tissues were obtained from patients who underwent hepatectomy consecutively in a single group at the Department of Hepatobiliary Surgery, Sun Yat-sen Cancer Center, between January 2001 and December 2006 and who fulfilled the following criteria: (1) exclusive treatment with chemotherapy or radiotherapy before tumour excision, (2) no evidence of concomitant extrahepatic disease, (3) no simultaneous use of local treatment modalities (i.e., radiofrequency ablation, microwave ablation). The patients included 116 males (90.6%) and 12 females (9.4%), with a median age of 50 years (range 23-79 years). Tumour size ranged from 1.2 to 19.0 cm, with a median of 6.0 cm. All tumours were histologically diagnosed as HCC with Edmondson-Steiner grade I in 8 cases, grade II in 61, grade III in 54, and grade IV in 5. The tumour stages were classified according to the 6th Edition tumour-node-metastasis (TNM) classification of the International Union Against Cancer [23]. Sixty-seven cases were classified as stage I, 26 as stage II, and 35 as stage III. In this study, non-tumorous tissues adjacent to tumour (NT) were defined as 2.0 cm from the margin, which were confirmed negative by histological examination, as used in other studies[24,25]. Twenty normal non-cirrhotic liver tissue samples (N), which were from patients with liver haemangioma or focal nodular hyperplasia, were included as controls. All specimens were obtained immediately after surgical resection, snap-frozen in liquid nitrogen, and kept at -80°C until use. All recruited patients provided written informed consent before hepatectomy, and the study protocol was approved by the Ethics Committee of Sun Yat-sen University Cancer Center.

### Reverse-transcription PCR analysis

All samples were evaluated in a blinded reverse-transcription polymerase chain reaction (RT-PCR) procedure without knowledge of the clinicopathological or follow-up data until the PCR results were finalised. PCR was performed as described previously [26]. Briefly, total RNA was isolated with TRIzol-A^+ ^agent and treated with DNase I (Invitrogen Inc., Carlsbad, CA) to remove DNA contamination. Reverse transcription was performed with the SuperScript RT kit (Promega Inc., Madison, WI) following the manufacturer's instructions. The cDNA templates were subjected to PCR amplification. The reaction conditions for *MACC1 *were as follows: pre-denaturation at 94° for 5 min; 34 cycles of denaturation at 94° for 30 s, annealing at 60° for 30 s and extension at 72° for 30 s; and final extension at 72° for 10 min. The final products were analysed by 2.0% agarose gel electrophoresis and stained with ethidium bromide. To detect any potential genomic DNA contamination, PCR reactions were also performed in RNA control samples that lacked reverse transcriptase during cDNA synthesis. Each PCR was performed in duplicate.

### Real-time quantitative PCR analysis

Real-time quantitative PCR (Q-PCR) was performed in the same batch of cDNA prepared for RT-PCR to quantify *MACC1 *mRNA. Moreover, to explore the correlation of *MACC1 *and c-MET, c-MET mRNA was also quantified in 128 paired tumour specimens. The primer sets were 5'-TTCTTTTGATTCCTCCGGTGA-3' (forward) and 5'-ACTCTGATGGGCATGTGCTG-3' (reverse) for *MACC1*; 5'-GGATGCGTGCATTTATCAGA-3' (forward) and 5'-GTTGATAGGGCAGACGTTCG-3' (reverse) for 18s rRNA; and 5'-GCTAAAATGCTGGCACCCTAA-3' (forward) and 5'-ATAGTGCTCCCCAATGAAAGTAGAGA-3' (reverse) for c-Met. Q-PCR was performed with an ABI Prism 7900 HT Sequence Detection System (Applied Biosystems) and SYBR green I Master Mix kit (Invitrogen). 18s rRNA was analysed to normalise Q-PCR data. The threshold cycle (Ct) value for triplicate reactions was averaged, and the relative genomic expression was calculated by 2^-ΔΔCt ^value [ΔCt = Ct (*MACC1*) - Ct (18s)] [27]. Melting curves were performed to ensure only a single product was amplified.

### Follow-up

The follow-up duration was defined as the interval between the date of operation and the date of death or last follow-up. The study was censored on 31 January 2010. The median follow-up time was 30.5 months, ranging from 3 to 85 months. All patients were followed up every 1-3 months in the first year and every 3-6 months thereafter. The follow-up protocol included physical examination, serum alpha-fetoprotein (AFP) level, chest X-ray, and abdominal ultrasonography. Computed tomography and/or magnetic resonance imaging and/or positron emission tomography were performed when intrahepatic relapse or distant metastasis was suspected. During the course of follow-up, 64 of 128 patients (50.0%) were found with intrahepatic recurrence, 18 patients (14.1%) developed distant metastases, 49 patients (38.3%) died of cancer-related causes, and 79 patients (61.7%) were still alive.

### Statistical analysis

All statistical analyses were carried out using the SPSS 16.0 statistical software package (SPSS Inc., Chicago, IL). The chi-square test or Fisher's exact test was used to comparison of frequencies. Spearman's correlation test was applied to analyse the correlation. Overall and disease-free survival curves were generated using the Kaplan-Meier method, and the difference between curves was assessed by the log-rank test. Independent prognostic factors were estimated by the Cox proportional hazards stepwise regression model. All *P *values were 2-sided. A *P *value of less than 0.05 was considered statistically significant.

## Results

### *MACC1 *expression in human hepatoma cell lines and HCC tissues

**Figure 1:**
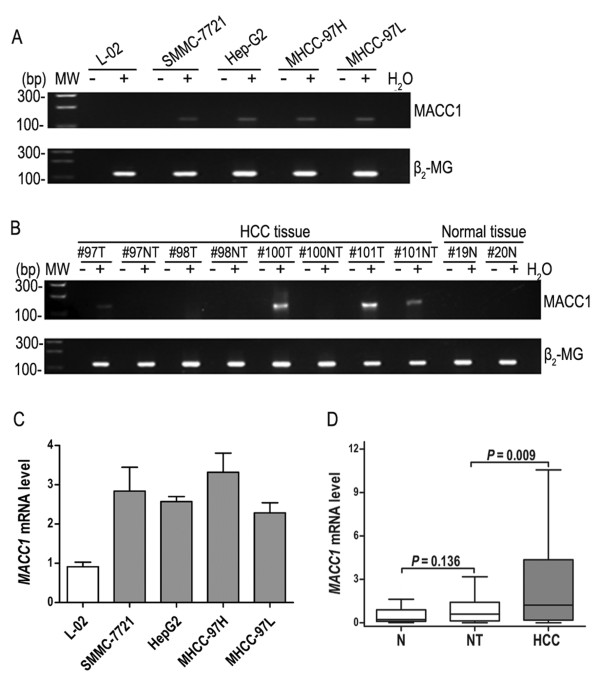
**Analysis of *MACC1 *expression in liver tissues and cell lines**. **A**: *MACC1 *expression in four human hepatoma cell lines (HepG2, SMMC-7721, MHCC-97H, and MHCC-97L) and one normal liver cell line, L-02, by reverse-transcription PCR (RT-PCR). **B**: Representative *MACC1 *mRNA expression in hepatocellular carcinomas (T) and matching non-cancerous (NT) and normal non-cirrhosis (N) liver tissue using RT-PCR. An RT-negative control (-) was added to rule out false positives resulting from contaminated DNA. Products of 136 bp were expected for *MACC1 *transcripts. **C**: Comparison of *MACC1 *expression levels in five liver cell lines, as determined by real-time quantitative-PCR (Q-PCR). **D**: Comparison of *MACC1 *expression levels in 20 normal liver tissues (N), and 128 paired HCC with matching non-cancerous (NT) specimens by Q-PCR. The differences among HCC tissues (median 1.632), the adjacent non-cancerous tissues (median 0.601), and normal tissues (median 0.218) are significant (*P *= 0.009, *P *= 0.008, respectively), and no significant difference was found between the adjacent non-cancerous and normal tissues (*P *= 0.136).

We first examined *MACC1 *expression in five liver cell lines. RT-PCR showed that there was a lack of *MACC1 *expression in the normal liver cell line L-02, while all of four hepatoma cell lines (SMMC-7721, Hep-G2, MHCC-97L and MHCC-97H) expressed *MACC1 *mRNA (Figure 1A). Of 128 paired HCC patients' specimens, the frequency of *MACC1*-positive expression was significantly higher in HCC tissues (53/128, 41.4%) than in the corresponding non-tumorous liver tissues (27/128, 21.1%, *P *< 0.001). The expression of *MACC1 *mRNA in normal non-cirrhotic liver tissues was undetectable (Figure 1B).

Similar results were obtained from the same batch of cell lines using Q-PCR, which indicated high *MACC1 *mRNA expression in liver cancer cell lines, while there was low expression in normal liver cells (Figure 1C). In 128 paired HCC patients' specimens, Q-PCR showed that *MACC1 *mRNA expression was higher in HCC tissues than in adjacent non-tumorous liver tissues or normal tissues (*P *= 0.009, *P *= 0.008, respectively) (Figure 1D). The dissociation curve showed a single peak as expected for *MACC1 *(data not shown). As we expected, Spearman's correlation test showed that the high-level expression of *MACC1 *detected by Q-PCR always indicated positive expression *MACC1 *according to RT-PCR (*r *= 0.223, *P *= 0.011).

### Correlation between *MACC1 *and c-MET mRNA expression

**Figure 2:**
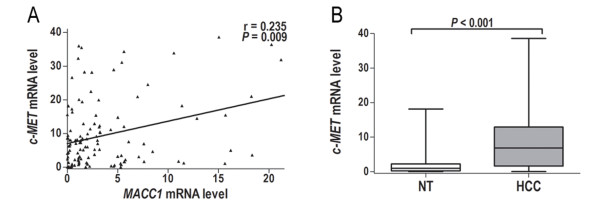
**c-MET expression and correlation with *MACC1 *in 128 paired HCC specimens: **A ***MACC1 *levels from 128 HCC specimens were plotted against c-MET levels from the same patients**. Linear regression analysis showed a significant positive correlation between *MACC1 *and c-MET (*r *= 0.567, *P *= 0.001). **B **Comparison of c-MET expression levels in 128 HCC specimens. The difference between HCC tissues and the adjacent non-cancerous tissues (NT) is significant (*P *< 0.001).

To address whether *MACC1 *and c-MET mRNA levels were correlated, Q-PCR was applied to compare them in 128 paired HCC specimens. When the expression levels of both *MACC1 *and c-MET were plotted on a scatterplot diagram, the positive relationship became clear (Figure 2A). There was indeed a significant positive correlation between the expression levels of *MACC1 *and c-MET (*r *= 0.235, *P *= 0.009, Spearman's correlation test). In addition, the expression level of c-MET was significantly higher in HCC tissues compared with adjacent non-cancerous tissues (*P *< 0.001) (Figure 2B).

### Correlation of *MACC1 *mRNA expression and clinicopathologic parameters

Next, to explore potential cutoff values for *MACC1*, a threshold value was chosen using a minimum *P *value approach as described by Mazumdar et al. [28]. In the multivariable setting, the level of *MACC1 *in HCC tissue that was associated with survival in terms of the minimum *P *value was 2.3-fold that of the non-cancerous tissue level. In the present study, 51 patients with 2.3-fold or more increased *MACC1 *in HCC tissues were defined as high-level, and 77 cases with below 2.3-fold expression were consider low-level. It should be mentioned that among 128 patients, 5 patients labelled as positive with conventional PCR were classified into the low group, while 3 *MACC1*-negative patients belonged to the high group. The slight inconsistency between Q-PCR and conventional RT-PCR results could have occurred because conventional PCR analysis is more inclined to be influenced by PCR reaction conditions. *MACC1 *mRNA expression was significantly associated with preoperative serum AFP level using Q-PCR analysis. High *MACC1 *expression was more frequent in high-AFP HCC patients (*P *= 0.025). No relationship was found between the expression of *MACC1 *and other clinicopathological variables, including gender, age, hepatitis B surface antigen (HBsAg) status, liver cirrhosis, TNM stage, tumour size, tumour number, tumour capsule, vascular invasion, and Edmondson-Steiner grade (Table 1).

**Table 1:** Correlations between *MACC1 *mRNA expression and clinicopathologic features of HCC

Characteristics	No. patients	*MACC1 *mRNA high level	*P *value*
Gender			
Female	12	58.3% (7/12)	0.169
Male	116	37.9% (44/116)	
Age (years)^†^			
≤ 50	65	44.6% (29/65)	0.263
> 50	63	34.9% (22/63)	
HBsAg status			
Negative	18	33.3% (6/18)	0.543
Positive	110	40.9% (45/110)	
AFP (μg/l)			
≤ 400	78	32.1% (25/78)	**0.025**
> 400	50	52.0% (26/50)	
Cirrhosis			
No	13	30.8% (4/13)	0.481
Yes	115	40.9% (47/115)	
Child-Pugh classification^‡^			
A	116	39.7% (46/116)	0.892
B	12	41.7% (5/12)	
Tumour size (cm)			
≤ 5	50	40.0% (20/50)	0.977
> 5	78	39.7% (31/78)	
Multiple tumours			
No	91	39.6% (36/91)	0.918
Yes	37	40.5% (15/37)	
Tumour capsule			
Complete	32	34.4% (11/32)	0.466
No/incomplete	96	41.7% (40/96)	
Vascular invasion			
No	98	37.8% (37/98)	0.383
Yes	30	46.7% (14/30)	
Edmondson-Steiner grade			
I/II	69	34.8% (24/69)	0.206
III/IV	59	39.8% (27/59)	
TNM stage			
I	67	31.3% (21/67)	0.077
II	26	42.3% (11/26)	
III	35	54.3%(19/31)	

*MACC1*, metastasis-associated in colon cancer-1; *HCC*, hepatocellular carcinoma; *AFP*, α-fetoprotein; *HBsAg*, hepatitis B surface antigen.

*Chi-square or Fisher's exact test.

^†^Patients were divided according to the median age.

^‡^No patients with Child-Pugh C were included.

### Prognostic of HCC subtypes defined by *MACC1 *level

**Figure 3:**
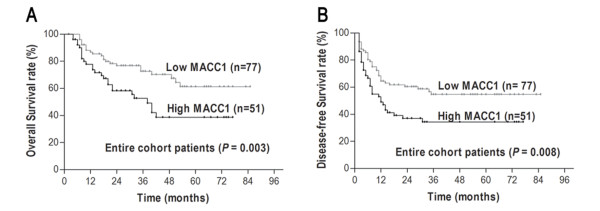
**Kaplan-Meier survival curves according to *MACC1 *expression in 128 HCC patients: **A **Overall survival (log-rank *P *= 0.003)**. **B **Disease-free survival (log-rank *P *= 0.008).

Significant OS and DFS advantages were observed for the patients with low *MACC1 *mRNA. The 5-year OS rate of the low-level group was 61.9%, which was significantly higher than that of the high-level group (37.6%, *P *= 0.003). The 5-year DFS rate of the low-level group was 54.5%, which was significantly higher than that of the high-level group (33.5%, *P *= 0.008) (Figure 3). The associations of OS and DFS with clinicopathological variables in our 128 cases of HCC are shown in Table 2. In a multivariate analysis model, *MACC1 *remained significantly associated with OS (HR 2.230; 95% CI, 1.257-3.957; *P *= 0.006) and DFS (HR 1.687; 95% CI, 1.034-2.751; *P *= 0.036) (Table 3). Low *MACC1 *indicates longer distant metastasis-free survival (MFS) for colon cancer patients[22]. However, no such correlation was found between *MACC1 *expression and MFS among these HCC patients (*P *= 0.803).

**Table 2:** Univariate prognostic analysis of overall survival and disease-free survival in HCC patients

Variable	Overall survival rate (%)			Disease-free survival rate (%)		
	
	3 y	5 y	*P *value	3 y	5 y	*P *value
Gender						
Female (*n *= 12)	91.3	61.8	0.328	66.7	66.7	0.176
Male (*n *= 116)	61.9	51.0		43.8	43.8	
Age (years)						
≤ 50 (*n *= 65)	62.5	52.2	0.567	42.7	42.7	0.101
> 50 (*n *= 63)	67.0	51.9		49.4	49.4	
HBsAg status						
Negative (*n *= 18)	88.5	62.1	0.122	71.8	71.8	**0.027**
Positive (*n *= 110)	60.5	49.7		41.9	41.9	
AFP (μg/l)						
≤ 400 (*n *= 78)	59.3	47.7	0.219	44.3	44.3	0.555
> 400 (*n *= 50)	75.0	59.8		50.9	50.9	
Cirrhosis						
No (*n *= 13)	91.7	80.2	0.053	54.9	54.9	0.184
Yes (*n *= 115)	61.3	48.1		44.6	44.6	
Child-Pugh classification						
A (*n *= 116)	65.4	55.0	0.378	48.8	48.8	0.154
B (*n *= 12)	58.3	29.2		20.8	20.8	
Tumour size (cm)						
≤ 5 (*n *= 50)	81.7	77.3	**< 0.001**	72.1	72.1	**< 0.001**
> 5 (*n *= 78)	52.6	36.3		29.2	29.2	
Multiple tumours						
No (*n *= 91)	70.0	60.4	**0.002**	56.5	56.5	**< 0.001**
Yes (*n *= 37)	49.3	30.2		19.8	19.8	
Tumour capsule						
Complete (*n *= 32)	71.2	71.2	0.165	61.1	61.1	0.096
No/incomplete (*n *= 96)	62.5	46.4		41.0	41.0	
Vascular invasion						
No (*n *= 98)	71.7	65.3	**< 0.001**	54.2	54.2	**< 0.001**
Yes (*n *= 30)	40.8	11.6		19.4	19.4	
TNM stage						
I (*n *= 67)	79.3	72.1	**< 0.001**	64.7	64.7	**< 0.001**
II (*n *= 30)	54.9	38.4		30.8	30.8	
III (*n *= 31)	42.0	22.5		20.7	20.7	
Edmondson-Steiner grade						
I/II (*n *= 69)	75.9	63.0	**0.004**	56.7	56.7	**0.003**
III/IV (*n *= 59)	50.6	38.4		33.4	33.4	
*MACC1 *expression level						
Low (*n *= 71)	73.5	61.9	**0.003**	54.5	54.5	**0.008**
High (*n *= 57)	51.8	37.6		33.5	33.5	

*MACC1*, metastasis-associated in colon cancer-1; *HCC*, hepatocellular carcinoma; *AFP*, α-fetoprotein; *HBsAg*, hepatitis B surface antigen.

**Table 3:** Multivariate analysis of factors contributing to overall survival and disease-free survival in HCC patients

Variable	Overall survival		Disease-free survival	
	
	HR (95% CI)	*P *value	HR (95% CI)	*P *value
Tumour size	3.008 (1.315-6.881)	0.009	3.227 (1.664-6.260)	0.001
Multiple tumours	1.361 (0.614-3.017)	0.447	2.254 (1.175-4.326)	0.014
Vascular invasion	2.280 (1.140-4.561)	0.020	1.873 (1.002-3.545)	0.049
HBsAg status	-	-	2.922 (1.153-7.403)	0.024
Edmondson-Steiner grade	1.853 (1.016-3.379)	0.044	1.615 (0.964-2.705)	0.068
TNM stage	1.136 (0.650-1.985)	0.654	0.865 (0.546-1.369)	0.535
*MACC1 *expression level	2.230 (1.257-3.957)	0.006	1.687 (1.034-2.751)	0.036

*MACC1*, metastasis-associated in colon cancer-1; *HCC*, hepatocellular carcinoma; *HBsAg*, hepatitis B surface antigen; *HR*, hazard ratio; *CI*, confidence interval.

### Stratified univariate and multivariate analysis

**Figure 4:**
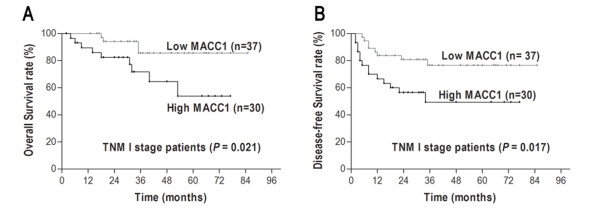
**Kaplan-Meier curves according to *MACC1 *expression in TNM stage I HCC patients**: **A **Overall survival (log-rank *P *= 0.021), **B **Disease-free survival (log-rank *P *= 0.017).

Because survival might be associated with the pathological TNM stage, we stratified the data according to TNM stage and investigated the prognostic value of *MACC1 *in different stages. For the 67 TNM stage I patients, significant correlations were found between *MACC1 *expression and OS (*P *= 0.021) and DFS (*P *= 0.017) (Figure 4). *MACC1 *had no prognostic value regarding OS or DFS for patients with TNM stage II or III (all *P *> 0.05). The associations of OS and DFS with clinicopathological features in TNM stage I HCC are shown in Table 4. In the Cox model adjusting for other prognostic variables, *MACC1 *was an independent negative prognostic factor for survival in TNM stage I patients (Table 5). Patients with high *MACC1 *expression had poorer OS (HR 2.643; 95% CI, 1.103-6.329; *P *= 0.029) and DFS (HR 3.316; 95% CI, 1.012-10.859; *P *= 0.048) than those with low *MACC1 *expression in TNM stage I.

**Table 4:** Univariate prognostic analysis of overall survival and disease-free survival in TNM stage I HCC patients

Variables	Overall survival rate (%)			Disease-free survival rate (%)		
	
	3 y	5 y	*P *value	3 y	5 y	*P *value
Gender						
Female (*n *= 9)	100.0	81.8	0.438	77.8	77.8	0.473
Male (*n *= 58)	75.7	71.1		62.3	62.3	
Age (years)						
≤ 50 (*n *= 32)	87.4	80.4	0.213	71.3	71.3	0.560
> 50 (*n *= 35)	73.0	65.7		59.4	59.4	
HBsAg status						
Negative (*n *= 13)	83.9	83.9	0.752	76.2	76.2	0.426
Positive (*n *= 54	78.2	69.4		62.1	62.1	
AFP (μg/l)						
≤ 400 (*n *= 43)	71.0	65.3	0.199	62.1	62.1	0.690
> 400 (*n *= 24)	91.1	82.8		68.0	68.0	
Cirrhosis						
No (*n *= 8)	100.0	100.0	0.128	75.0	75.0	0.565
Yes (*n *= 59)	76.0	67.5		63.2	63.2	
Child-Pugh classification						
A (*n *= 61)	78.9	75.5	0.499	67.2	67.2	0.519
B (*n *= 6)	83.3	55.6		44.4	44.4	
Tumour size (cm)						
≤ 5 (*n *= 40)	82.6	82.6	0.061	77.7	77.7	0.006
> 5 (*n *= 27)	73.0	57.0		45.7	45.7	
Tumour capsule						
Complete (*n *= 26)	81.0	81.0	0.787	67.3	67.3	0.957
No/incomplete (*n *= 41)	79.1	68.3		63.3	63.3	
Edmondson-Steiner grade						
I/II (*n *= 46)	80.9	80.9	0.172	68.3	68.3	0.338
III/IV (*n *= 21)	73.7	60.0		57.1	57.1	
*MACC1 *expression level						
Low (*n *= 37)	85.6	85.6	0.021	76.5	76.5	0.017
High (*n *= 30)	71.1	53.3		49.4	49.4	

*MACC1*, metastasis-associated in colon cancer-1; *HCC*, hepatocellular carcinoma; *AFP*, α-fetoprotein; *HBsAg*, hepatitis B surface antigen.

**Table 5:** Multivariate analysis of factors contributing to overall survival and disease-free survival in TNM stage I HCC patients

Variable	Overall survival		Disease-free survival	
	
	HR (95% CI)	*P *value	HR (95% CI)	*P *value
Tumour size	3.062(1.280-7.323)	0.012	2.445(0.794-7.532)	0.119
*MACC1 *expression level	2.643(1.103-6.329)	0.029	3.316(1.012-10.859)	0.048

*MACC1*, metastasis-associated in colon cancer-1; *HCC*, hepatocellular carcinoma; *HR*, hazard ratio; *CI*, confidence interval.

## Discussion

The transcript levels of *MACC1 *in normal liver tissue are 14 × 10^6^, as detected by expressed sequence tags (ESTs), compared with 20 × 10^6 ^in malignant liver tissue, according to the EST profile viewer of the NCBI UniGene database http://www.ncbi.nlm.gov/UniGene. These were supported by our study and another paper published recently. Shirahata et al. [29] showed that *MACC1 *expression was significantly correlated with vascular invasion and serum AFP level. However, with their small number of HCC patients (n = 30), statistical power was limited, and the authors did not explore its clinical predictive value for HCC patients. In this study, we analysed the mRNA expression of *MACC1 *in a relatively large population of HCC patients and correlated it with clinicopathological features and prognosis to determine whether this biomarker could predict disease outcomes. *MACC1 *expression in HCC tissue was significantly higher than in nonmalignant tissue. Importantly, high *MACC1 *expression was significantly correlated with more aggressive behaviour in terms of shorter OS and DFS and higher serum AFP, which is a putative clinicopathologic marker of HCC invasiveness and unfavourable prognosis [30]. These data indicate that high *MACC1 *expression occurs in HCC and is associated with an aggressive invasion phenotype. Although elevated *MACC1 *expression was associated with high AFP in our study, which agrees with the data of Shirahata et al., no significant correlation has been observed between vascular invasion and *MACC1 *expression. This disparity presumably stems from different sample sizes and heterogeneity of study populations, which led to the relatively low proportion (4/30) of vascular invasion in the study of Shirahat's group [29]. Stein et al. observed that high *MACC1 *levels indicated poor MFS for colon cancer. In contrast to colon cancer, intrahepatic metastasis is the most frequent pattern in the progression of HCC [5,31], which may partially explain why no such relationship was found in this study. However, it is difficult to define whether intrahepatic recurrence after hepatectomy originates from either residual intrahepatic metastasis or metachronous multicentric carcinogenesis [32,33]. Because the number of extrahepatic metastasis cases was limited in this study (n = 18), future studies are needed to address this issue more definitively.

Because MACC1 may promote cell migration and invasion by upregulating the downstream c-MET gene in colon cancer [22], we sought to determine whether such a mechanism might contribute to the increased invasiveness of HCC induced by MACC1. This study found a positive association between *MACC1 *and c-MET mRNA levels in HCC. This might support the existence of a regulatory and functional relationship between MACC1 and c-MET [34,35]. Furthermore, based on c-MET contributes to the aggressiveness of HCC [17], this correlation might further indicate that MACC1 enhances the invasiveness of liver cancer cells.

Our study shows that *MACC1 *was expressed highly in HCC samples and cultured cancer cell lines. The mechanism of this high expression is still unclear, but it is interesting to note that chromosome region 7p21, which contains the *MACC1 *gene [36], frequently exhibits aberrant amplification of expression in HCC [37-39].

We hypothesised that high expression of *MACC1 *was causally associated with HCC invasion, based on several lines of evidence. First, we have shown that high *MACC1 *expression in human HCC was associated with higher serum AFP and shorter OS and DFS. Second, the level of *MACC1 *mRNA was high in MHCC-97H cells (Figure 1C), which are derived from the high-metastasis-potential cell line MHCC97 [40]. Third, the level of *MACC1 *was positively correlated with that of c-MET, which plays critical roles in cancer cell migration and metastasis. Because this study enrolled mostly hepatitis B virus-dependent HCC patients (85.9%), it remains to be studied whether *MACC1 *as a marker of aggressive phenotype can be extended to HCC cases resulting from other aetiologies. Hepatitis virus-dependent and alcohol-dependent HCC display overexpression of MYC, whereas nonalcoholic steatohepatitis may evolve into malignancy via a MYC-independent mechanism, indicating that the existence of genetic discrepancies may occur via different aetiological routes [41]. Whether *MACC1 *is associated with certain aetiological factors needs to be further investigated.

Clinical stage is the most important factor influencing the prognosis of HCC patients. Several systems are available to classify HCC. Among them, the International Union Against Cancer's TNM staging is one of the most prevalent. Although the TNM system has successfully graded patients on their prognosis according to clinicopathological variables, it has reached its limit in providing critical information that may influence treatment strategy. It is difficult for liver surgeons to predict exactly which individuals will experience relapse among early-stage patients who have undergone curative treatment. To overcome the limitations of these traditional systems, many molecular markers have been investigated and shown to have potential predictive significance. However, to date, biomarkers that could stratify HCC patients with curative excision in TNM stage I are still substantially limited. In our stratified analysis, we found that *MACC1 *mRNA expression had clear prognostic value for OS and DFS in TNM stage I patients. These data imply that *MACC1 *mRNA might act as a predictive tool to identify patients with TNM stage I at high risk of recurrence.

MACC1 may act as a key regulator of the HGF/c-MET pathway, leading to distant metastases in colon cancer [22]. The relationships of MACC1 with other signalling molecules and pathways must be further evaluated to better understand the molecular pathogenesis of these tumours and develop more effective targeted therapeutic strategies.

## Conclusion

This study established a correlation between *MACC1 *expression and HCC prognosis. *MACC1 *was highly expressed in HCC tissues and predicted the prognosis of HCC patients, suggesting MACC1 may be involved in the HCC malignant process. This information can be used to identify high-risk HCC patients who may benefit from more intensive treatment and follow-up care after resection of primary tumours. It also justifies further studies to gain insight into the underlying biology of MACC1 and to strive for a positive response after administration of innovative therapies targeted at MACC1.

## List of abbreviations

HCC: hepatocellular carcinoma; RT-PCR: reverse-transcription polymerase chain reaction; Q-PCR: real-time quantitative PCR; cDNA: complementary DNA; MACC1: metastasis-associated in colon cancer-1; HGF: hepatocyte growth factor; c-MET: c-MET proto-oncogene; AFP: alpha fetoprotein; TNM: tumour-node-metastasis; OS: overall survival; DFS: disease-free survival; MFS: metastasis-free survival.

## Competing interests

The authors declare that they have no competing interests.

## Authors' contributions

YFY and JPW were responsible for the design of this study. JLQ conducted the experiments and drafted the manuscript. PZH participated in the data analysis. CLL and LW helped in sample collection. QL, JH and BKL helped in amending the manuscript. All authors read and approved the final manuscript.
